# Finite Volume Effects in Water Nanodroplets: A Molecular Level Investigation

**DOI:** 10.1021/acsnano.5c04422

**Published:** 2025-06-24

**Authors:** Li Zhang, Saranya Pullanchery, Paul S. Cremer, Sylvie Roke

**Affiliations:** a Laboratory for Fundamental BioPhotonics, Institute of Bioengineering (IBI), School of Engineering (STI), 27218 École Polytechnique Fédérale de Lausanne (EPFL), CH-1015 Lausanne, Switzerland; b Institute of Materials Science and Engineering (IMX), School of Engineering (STI), 27218 École Polytechnique Fédérale de Lausanne (EPFL), CH-1015 Lausanne, Switzerland; c Lausanne Centre for Ultrafast Science, 27218 École Polytechnique Fédérale de Lausanne (EPFL), CH-1015 Lausanne, Switzerland; d Department of Chemistry, 8082 Pennsylvania State University, University Park, Pennsylvania 16802, United States

**Keywords:** water, hydrogen bonding, emulsions, sum frequency generation, vibrational spectroscopy, interfaces, droplets

## Abstract

Aqueous interfaces are responsible for a plethora of processes. At the nanoscale, interfaces are overwhelmingly influenced by finite volume effects that are thought to impact both molecular level and macroscopic properties. Here, finite volume effects are investigated using electrophoretic mobility and vibrational sum frequency scattering of water nanodroplets in oil and oil nanodroplets in water, made from the same chemicals. Notably, there is a substantial difference in the orientational ordering of water between the two aqueous interfaces. Isotope dilution studies reveal that water outside the oil droplets participates significantly in intramolecular coupling, while water inside the droplets predominantly exhibits intermolecular coupling. These spectral variations underlie different water structures, pointing to a larger heterogeneity inside water droplets, which are explained by finite volume effects that include a pronounced difference in electrostatics.

## Graphical abstract

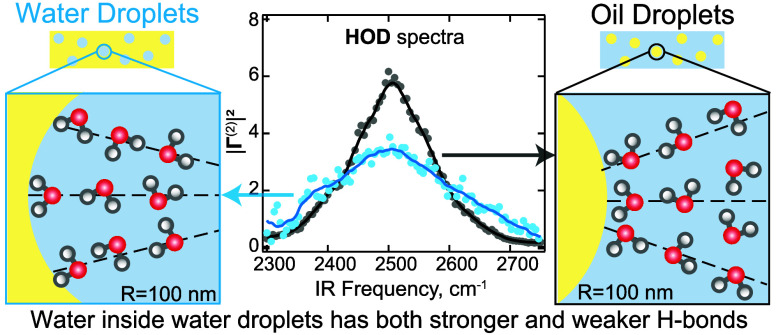

## Introduction

Finite volume effects refer to interactions under confinement or ramifications of such interactions that are different on the nanoscale. These effects are responsible for drastic changes in physical properties, including enabling the design of nanomaterials with specialized functions. For example, nanopores may induce freezing,^1,2^ and water between graphene sheets displays nonbulk-like behavior that is presently not well understood.^3,4^ Properties such as diffusion, viscosity, the dielectric function, water hydrogen bond structure and dynamics, thermal conductivity, and freezing can all be impacted by nanoscale confinement.^4–15^ Hydrophobic assembly is also strongly size-dependent,^16^ and local corrugation can have a major influence on solvation.

The restriction of a spatial dimension creates a different balance of interactions, such as electrostatic, hydrogen (H)-bonding, and other chemical interactions, as well as entropy in the available configuration space. Understanding finite volume effects in aqueous systems is a major challenge because it requires a deep understanding of individual molecular interactions and how they are manifested on vastly different length scales. Electrostatic fields on either side of a charged planar interface attenuate along the surface normal into the solution as predicted by the Poisson–Boltzmann equation,^17^ which depends on the dielectric constant of the media, and the ionic strength among other things. The exact results, however, also depend on the geometry of the interface, because the boundary conditions differ.^18,19^ Therefore, the electrostatic potential and electrostatic field is significantly different inside a droplet, around a droplet, or on a planar extended interface. This is illustrated in Figure 1, where the electrostatic field lines (Figure 1A–C) are drawn. The potential (Figure 1D–F), and its derivative, the electrostatic field, are plotted (Figure 1G–I) for three different configurations of the same materials: the planar oil–water interface (Figure 1A,D,G), 100 nm radius oil droplets in water (Figure 1B,E,H), and 100 nm radius water droplets in oil (Figure 1C,F,I). These plots were calculated with dielectric constants of ϵ = 78 and ϵ = 2 for water and oil respectively, an ionic strength of 1 μM, and a surface potential of −50 mV. The surface potential is chosen to be similar to what was previously measured for the oil-droplet-in-water interface.^20^ The ionic strength of the aqueous phase was set to 1 μM to represent experimental conditions (D_2_O typically has slightly higher conductivity than ultrapure water, and trace amounts of CO_2_ may dissolve during sample preparation). Figure 1 shows that, even with all relevant parameters being equal, the electrostatics operate in strikingly different ways. For example, at a planar extended oil–water interface, the electric field attenuates as it progresses into the aqueous solution due to dielectric and ionic screening but without having an electrostatic field divergence (Figure 1A). Around an oil droplet, the electrostatic field diverges, which is accompanied by a faster potential decay (Figure 1B) and thus a higher electrostatic field magnitude. Finally, inside water droplets (Figure 1C), the field lines are initially along the normal direction, and the field likely vanishes at the center of the nano-object, to avoid the creation of singularities. In this case the electrostatic potential remains nearly constant (Figure 1F) and changes very little throughout the droplet, leading to a much weaker electric field (Figure 1I). Thus, the electrostatic field experienced in the three aqueous phases is markedly different. Moreover, the behavior of salt ions is affected by finite volume effects as well,^19^ which can be expected to influence interfacial properties.

**Figure 1:**
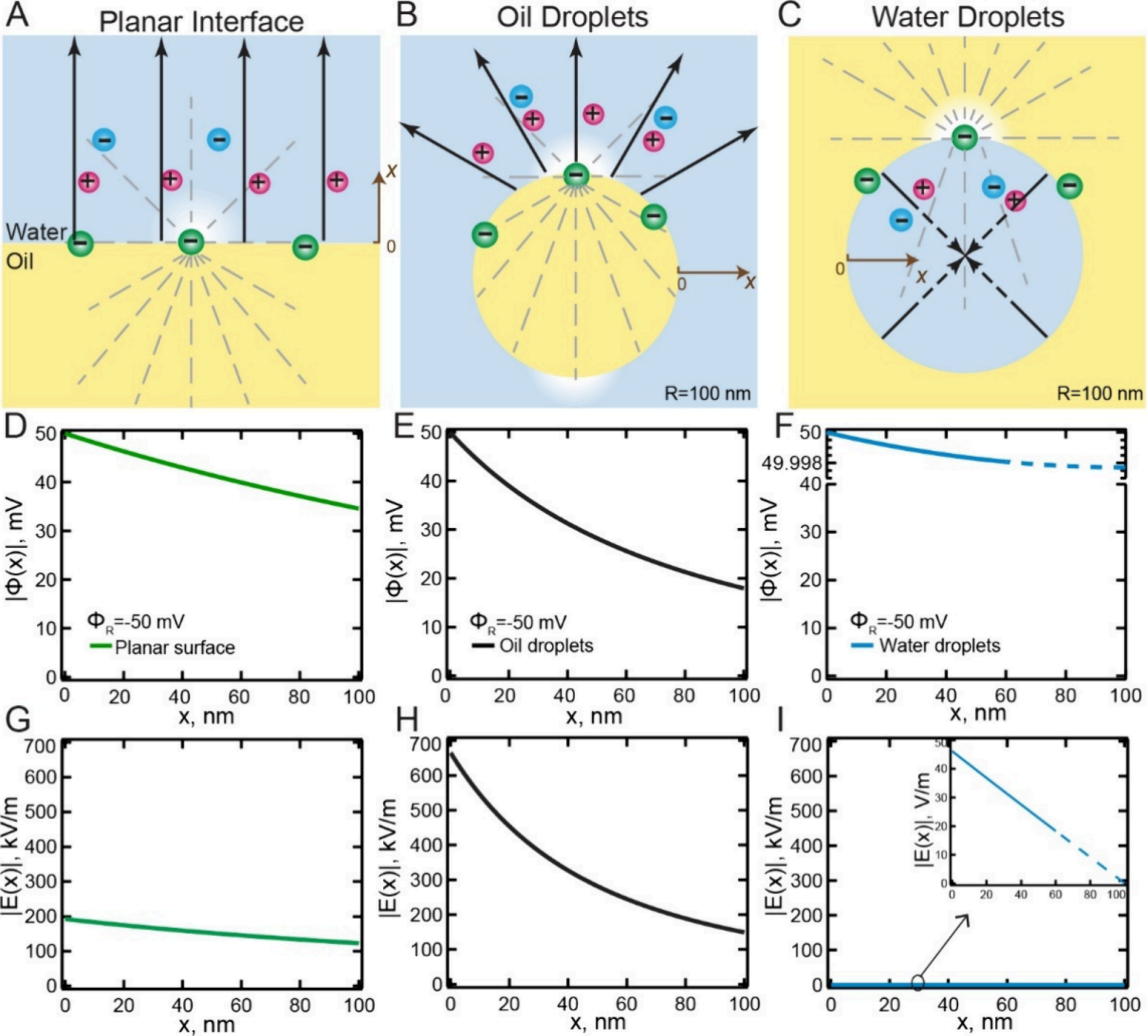
Electrostatic field for three different surface configurations. Schematic of the electrostatic field lines (A) at a planar oil/water interface, (B) around oil droplets, and (C) inside water droplets. The gray dashed lines are field lines from a single surface charge, the black solid lines with arrows illustrate averaged field lines that propagate from charged surfaces into the bulk solution, and the brown arrow labeled ‘*x*’ indicates the direction of the surface normal. (D–F) Equivalent electrostatic potential plots for three different configurations of the same materials all having an (arbitrary) surface potential of −50 mV and an ionic strength of 1 μM. For the dielectric constant (ϵ), the values of ϵ = 78 (water) and ϵ = 2 (oil) were taken. (D) Planar oil–water interface (green), (E) 100 nm radius oil droplets in water (black), and (F) 100 nm radius water droplets in oil (blue). Note the difference in scale between panels D, E, and F. Water droplets in oil show hardly any decay of the potential. (G-I) Magnitude of the electrostatic field |**E**|, as a function of *x* (|*d*Φ/*dx*|), for the same systems.

Recent studies comparing charged surfactant oil–water interfaces of oil nanodroplets with planar interfaces showed marked differences that can be explained by variations in the electrostatic balance of interactions: When charged oil droplets or other materials with a relatively low dielectric constant are dispersed in water, the charges on the surface of the droplets are only very slightly screened by the interior oil phase (see Figure 1B) and a collective repulsive interaction remains that drastically limits the surfactant concentration as observed previously.^21–23^ As such, this geometry imparts droplet stability even at low surface charge densities.^23^ The above-mentioned studies were performed using vibrational sum frequency scattering (SFS) which is a unique surface-sensitive technique to probe molecular structure 2–3 molecular dimensions deep into the nanodroplet interface.^22,24,25^ In SFS, femtosecond broadband infrared (IR) laser pulses are spatially and temporally overlapped with narrowband visible pulses inside a sample to generate sum frequency (SF) photons (Figure 2A). The SF response from submicron sized particles originates from the second- and third-order particle susceptibility, Γ^(2)^ and Γ^(3)^ respectively, which is nonzero only in regions of molecular anisotropy. Because of this, only molecules at the nanodroplet interface (via the Γ^(2)^ term) are probed, where spatial isotropy is broken. Such anisotropy arises from chemical interactions like H-bonding, or when electrostatic field interactions change the orientational distribution of dipolar molecules such as at aqueous interfaces (via the Γ^(3)^ term).^26–28^ Whether electrostatically induced finite volume effects have consequences for water in confined spaces (Figure 1C) has not been investigated yet.

**Figure 2:**
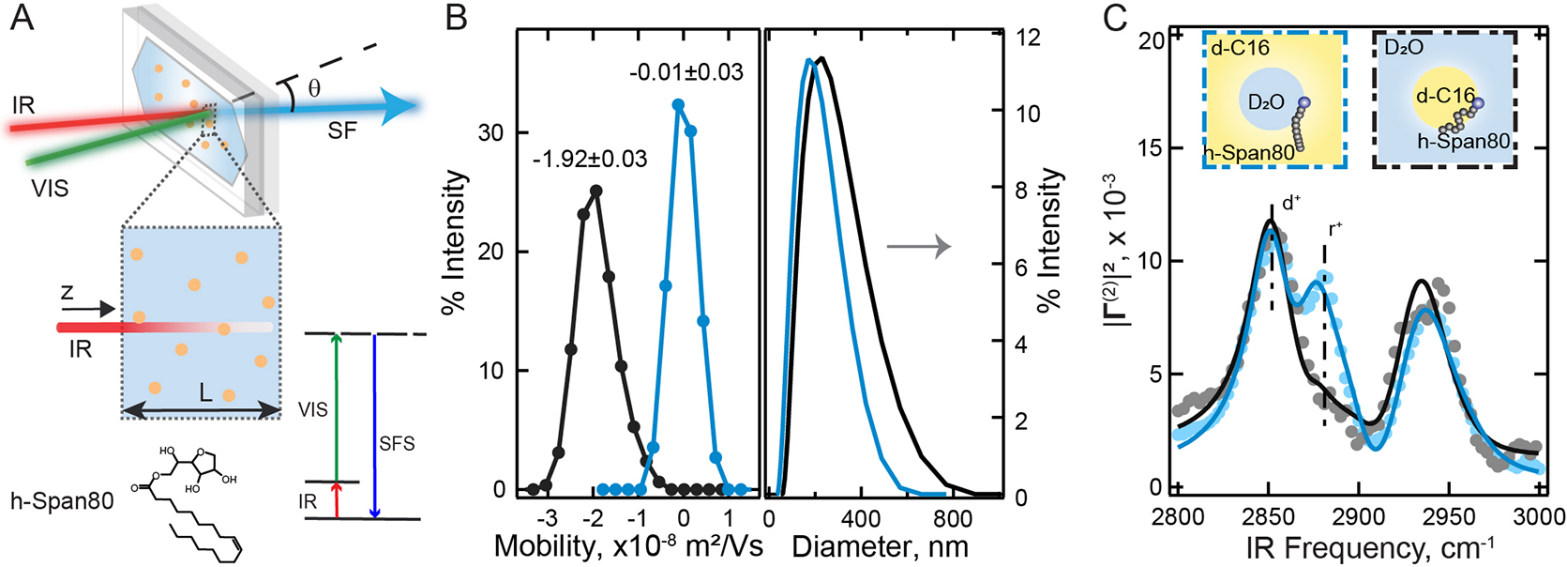
Oil-in-water versus water-in-oil nanodroplets. (A) Schematic of the vibrational SFS experiment (for oil droplets dispersed in water). The zoomed-in view of the sample cross-section shows the attenuation of the IR beam as it travels through the sample cell. The energy level diagram of vibrational SFS is shown at the bottom right. The chemical structure of the Span80 molecule is shown at the bottom left. (B) Recorded distributions of electrophoretic mobility (left) and diameter (right) for water-in-oil (blue) and oil-in-water droplets (black). The values reported above the mobility distribution curves correspond to the mean and standard deviation of the distribution. (C) Interfacial C–H stretch SF spectra of Span80 on the surface of water-in-oil droplets (blue) and oil-in-water droplets (black). The insets show schematic diagrams of water-in-oil and oil-in-water droplet surface structures. Both samples were prepared with 10 mM Span80 in the oil phase as discussed in the text. The solid lines are fits using eq S2, see the Supporting Information for details. The dashed lines represent the positions of d^+^ and r^+^ modes. All the spectra were recorded using the SSP polarization combination and were normalized to the unit area.

Hydrogen bonds and other chemical interactions that are directional and cooperative depend on the local three-dimensional (3D) surrounding structure. Vibrational dynamics of water have been found to be heterogeneous in water that is confined within reverse micelles, with the interfacial water showing slower dynamics than in the bulk.^29–32^ 3D confinement effects on the H-bond network of water were investigated in previous studies over larger length scales ranging from ∼60 to 150 nm, using zwitterionic liposomes of different sizes as confining entities. The H-bond network configuration was shown to be influenced by long-range confinement effects, which were different for light and heavy water, with H_2_O displaying confinement effects over much larger distances (>140 nm, involving >4 × 10^7^ H_2_O molecules) than D_2_O (<60 nm, involving (<3 × 10^6^ D_2_O molecules).^33^ The question as to what happens on the molecular level to the structure of water as a consequence of finite volume effects, and how this relates to macroscopic observables is still open. This is mainly because a direct experimental comparison of systems with inverse geometries, that is nano-objects in a main phase with materials in both phases inversed, has not yet been performed.

Herein, we examine the structure of water at the interface of oil nanodroplets in water and water nanodroplets in oil prepared using the same three chemicals (hexadecane, Span80, and water), with the aim to understand finite volume effects in water on the molecular level. We combine electrophoretic mobility measurements with vibrational SFS, with and without vibrational decoupling. Both droplet systems exhibit electrophoretic mobility. The vibrational interfacial water spectra in both systems are insensitive to the surfactant, which only partially covers the interface. The interfacial SF response from water inside the water droplet has markedly more low-frequency contributions than water surrounding the oil nanodroplet, revealing a notable difference in the degree of orientational ordering. Isotope dilution studies display further striking differences: water outside oil droplets significantly participates in intramolecular coupling, which changes the water spectrum through both broadening and shape changes. For the water droplets, no such coupling is detected. Instead, the spectrum is defined by intermolecular coupling, which leads to stronger H-bonding and a markedly red-shifted spectrum. These spectral differences in coupling highlight distinct water structures on water-in-oil and oil-in-water droplets, which are explained by differences induced by finite volume effects.

## Results and Discussion

### Droplets Inside-Out: Electrophoretic Mobility and Electrostatics

Water-in-oil droplets (W/O) and oil-in-water (O/W) droplets (nanoemulsions) were examined in the presence of Span80 as a surfactant (Figure 2A). Both stock systems have a 2 vol % droplet phase and a 98 vol % main phase. Span80, whose molecular structure is shown at the bottom left of Figure 2A, is an oil soluble surfactant, and its concentration in the oil phase was maintained at 10 mM for both nanoemulsion systems to ensure that the interfaces possess comparable chemical compositions. The nanodroplets were prepared using ultrasonication and had mean radii between 88–117 nm, with polydispersity indexes (PDIs) varying between 0.1–0.3 (see Table S1 for all droplet parameters). The size (diameter) distributions of water and oil droplets are plotted in Figure 2B (right). The measured electrophoretic mobility (μ) distributions for the two droplet systems are shown in Figure 2B (left), where the average mobility of the Span80 covered oil-in-water droplets was −1.92 ± 0.03 × 10^–8^ m^2^/(V s), and that of the Span80 covered water-in-oil droplets was −0.01 ± 0.03 × 10^–8^ m^2^/(V s). The difference of these values reflects the fact that water droplets, on average, move ∼200× slower in oil than oil droplets in water. This should also arise from the small (close to 0) net charge just outside the water droplets, as the oil phase is insulating with very little capacity to solvate ions that are not directly at the oil/water interface. It should be noted that the uncertainty in these measurements (±0.03 × 10^–8^ m^2^/(V s)) is the same, although the relative uncertainty in the latter case is far larger because its mean value is very close to zero.

The mobility (μ) and zeta (ζ-) potential are commonly related by Henry’s equation $\mu =\frac{\epsilon_{0}\epsilon \zeta f\left(\kappa R\right)}{\eta }$ (see Section S1 for details), where ϵ is the dielectric constant and η is the viscosity of the main phase. Converting the average mobility values into ζ-potential magnitudes for oil droplets (∼|46| mV) and water droplets (∼|29| mV), values are found to be of the same order of magnitude. Despite the significant difference in mobility this is caused by the far larger ϵ/η value of the oil. In addition, there is an effect of slip,^18^ which is different in both systems and likely influences the relationship between mobility and ζ-potential. However, this relation cannot be incorporated as the slip length is not known.

The electrophoretic mobility of droplets in water is determined by the interfacial charge, and the electric double layer properties of the inner/outer aqueous phase.^19^ While the chemicals are identical, the coupling between potential and droplet mobility follows different mechanisms with the inner and outer electric double layers playing different roles. This means that an oil droplet in water with a certain surface charge and ionic strength experiences a different mobility than a water droplet in oil that has the same surface charge and ionic strength. It will also have a different electrostatic field in the aqueous phase as was discussed in relation to Figure 1. In Figure 1E,H,F,I the decay of the electrostatic potential and field was computed using the solution to the linearized Poisson–Boltzmann equation (ref 19, eq 23). For oil droplets in water, the result is shown in Figure 1E,H, and for water droplets in oil the result is shown in Figure 1F,I. For the oil droplets in water, with a low ionic strength, the electrostatic field decays differently than at a planar oil–water interface (Figure 1D,G). Furthermore, for the case of water droplets in oil, the solution is again markedly different. In this case, for the same potential and ionic strength, the blue curve is obtained in Figure 1F,I. As can be seen, the electrostatic potential inside the droplet is nearly constant at all positions (note the adjusted *Y*-axis), resulting in a nearly vanishing electrostatic field. Both results depend heavily on the charge distribution inside the electric double layer. This, in turn, dramatically influences the interfacial water structure. Both can be characterized by vibrational sum frequency scattering (Figure 2A, refs 34–36 ). Below, we first investigate the molecular structure of Span80 (Figure 2C) and then the interfacial water (Figures 3 and 4).

**Figure 3:**
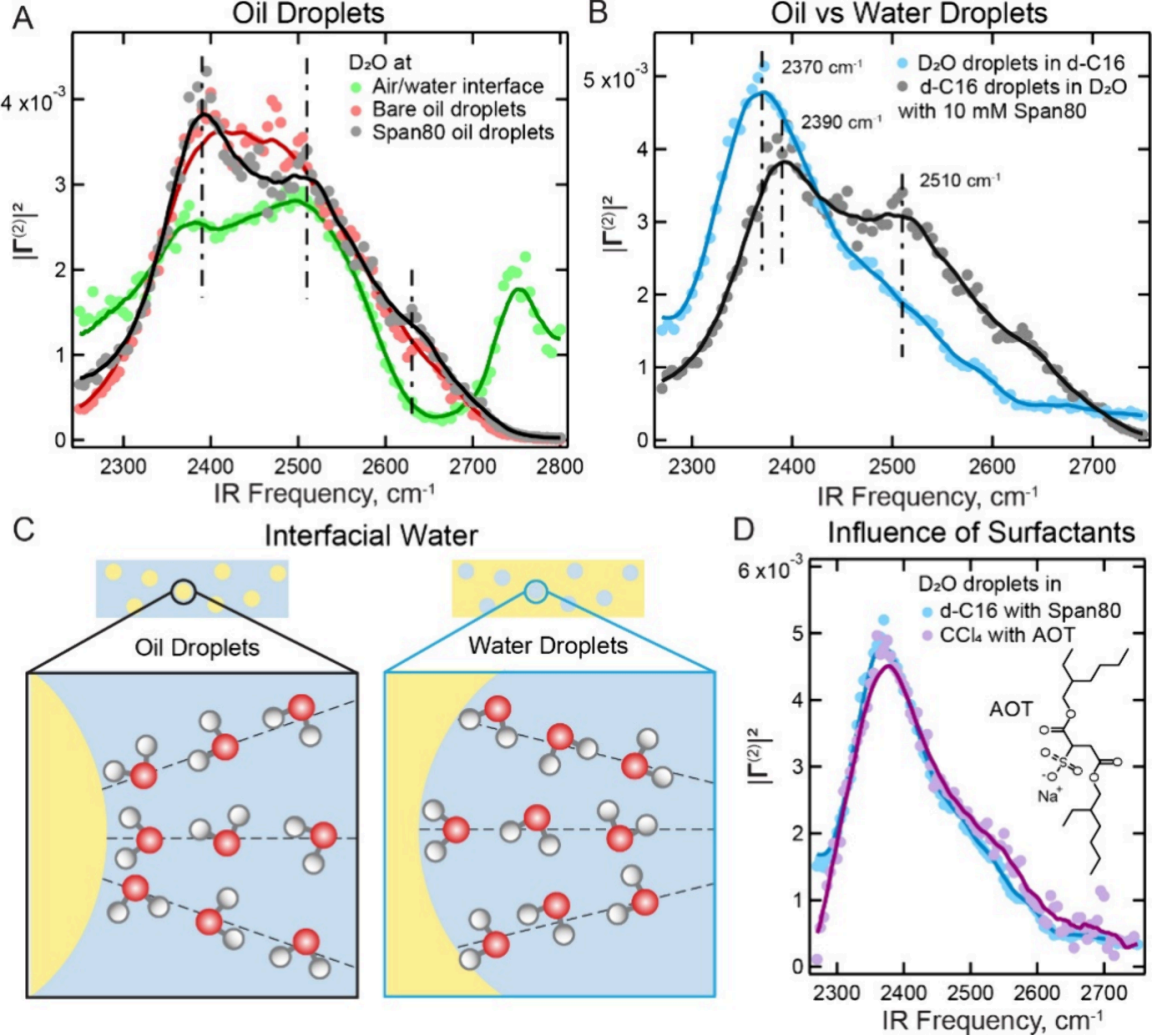
Water structure at the oil-in-water versus water-in-oil nanodroplets interface. (A) Interfacial O–D stretch SF spectra of air/water interface (green data, previously published in ref 36), bare hexadecane oil-in-water droplets (red data), and Span80-covered hexadecane oil-in-water droplets (black data). The dashed vertical lines represent the positions of the frequencies discussed in the text. The solid lines represent the running average as a guide to the eye. (B) Interfacial O–D stretch SF spectra of Span80-covered oil-in-water droplets (black data) and Span80-covered water-in-oil droplets (blue data). All Span80 containing droplet samples were prepared with 10 mM Span80 in the oil phase. The dashed lines represent the positions of the frequencies discussed in the text. The solid lines represent the running average as a guide to the eye. The spectra are averages of three measurements (see the Supporting Information, Figure S4 for the spectra with error bar). The spectra are normalized to the unit area to enable shape comparison. (C) Schematic diagrams of differences between the water structure outside oil droplets (left) and inside water droplets (right). (D) Interfacial O–D stretch spectra of AOT-covered water in CCl_4_ droplets (purple data) and Span80-covered water-in-oil droplets (blue data). The inset shows the chemical structure of AOT surfactant. All spectra were recorded using the SSP polarization combination. The spectra were corrected for infrared absorption by D_2_O or water droplets emulsion^25^ using the procedure described in the Supporting Information. The spectra are area normalized.

**Figure 4:**
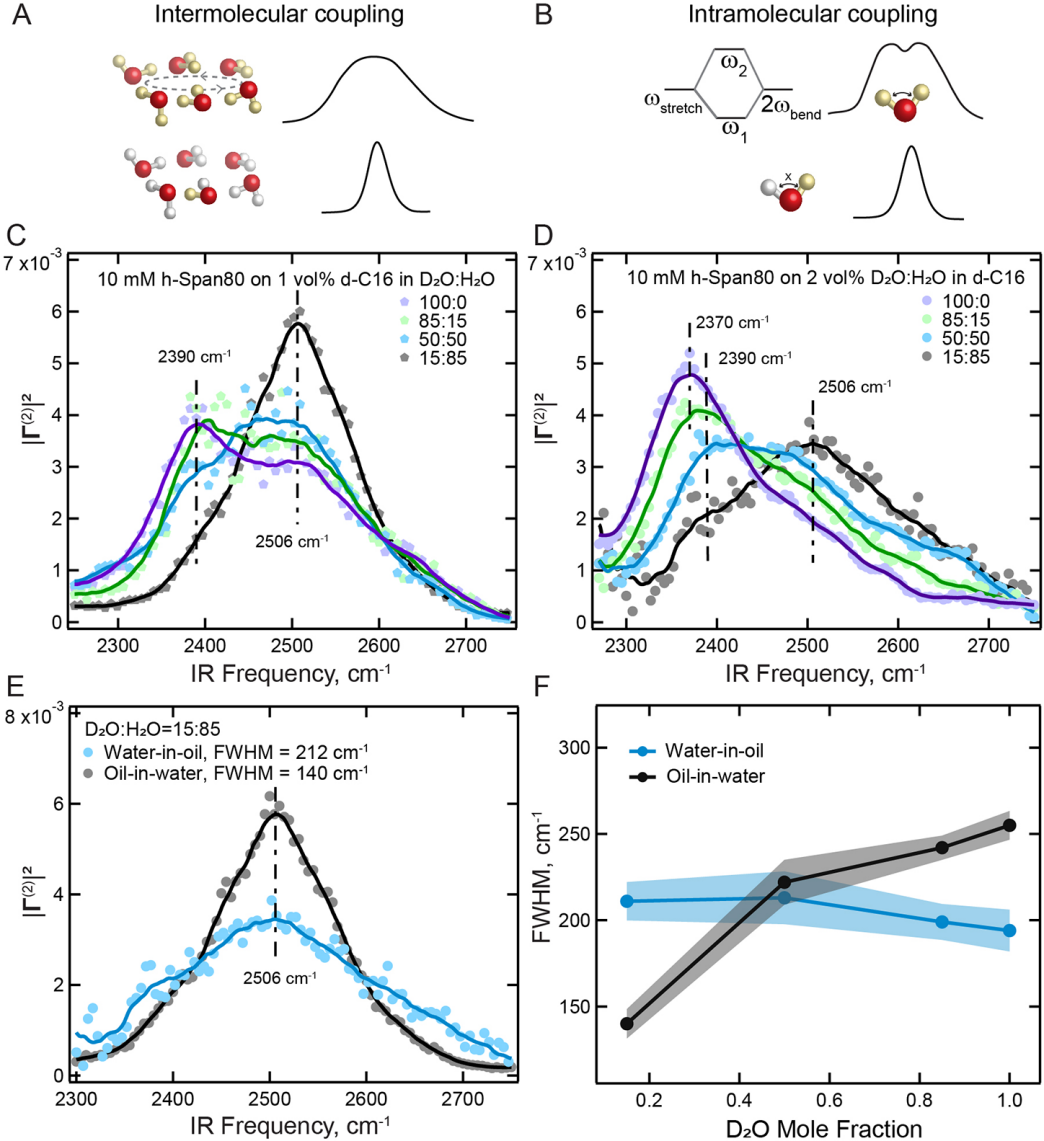
Disentangling intra- and intermolecular vibrational coupling. (A, B) Illustration of vibrational coupling effects. (A) Intermolecular coupling: when O–D modes are coupled to neighboring D_2_O molecules, the vibrational energy is delocalized onto several adjacent oscillators resulting in spectral broadening (top). Isotopic dilution turns off intermolecular coupling, resulting in spectral narrowing (bottom). (B) Intramolecular coupling: the symmetric stretch of D_2_O (ω_stretch_) and the overtone of the D_2_O bending mode (2ω_bend_) couple with each other, leading to the splitting of the O–D stretch band into ω_1_ and ω_2_ (top). Isotopic dilution results in HOD species, where the stretch and bend overtone modes are uncoupled, thus merging the two bands into a single peak (bottom). (C) O–D stretches SF spectra of Span80-covered oil-in-water droplet interfaces prepared using D_2_O/H_2_O mixtures. (D) O–D stretches SF spectra of Span80-covered water-in-oil droplet interfaces in which the water phase consists of different D_2_O/H_2_O mixtures. For both spectral data sets, the solid lines represent the running average and are guides to the eye. The vertical dashed lines represent the position of the strongly H-bonded O–D stretch modes and the uncoupled O–D mode in the case of 15% D_2_O. (E) Comparison of the area-normalized SF spectra of both inverse systems at a 15% volume ratio of D_2_O, having a mole percent of 0.02:0.25:0.73 for D_2_O:HOD:H_2_O. The spectra are averages of three measurements (see Supporting Information, Figure S4 for the spectra with error bar). All the spectra were recorded with the SSP polarization combination and are normalized to the unit area to enable the shape comparison. (F) Spectral fwhm for both emulsion systems at different D_2_O mole fractions.

### The Interfacial Structure of the Surfactant on Chemically Identical Droplet Interfaces

To distinguish between the SF responses of hexadecane and Span80, the oil phase was deuterated (n-C_16_D_34_, d-C16). Figure 2C shows the |Γ^(2)^|^2^ spectra of the C–H stretch modes of Span80 on the surface of oil-in-water droplets (black data) and water-in-oil droplets (blue data) measured using the SSP polarization combination, where the sum frequency and visible beams were S -polarized (i.e., polarized perpendicular to the scattering plane) and the IR beam was P-polarized (i.e., polarized parallel to the scattering plane). The vibrational modes at ∼2852 cm^–1^, ∼2875 cm^–1^, ∼2902 cm^–1^, ∼2920 cm^–1^, ∼2935 cm^–1^ and ∼2965 cm^–1^ correspond to the symmetric (s-) CH_2_ stretch mode (d^+^), the s-CH_3_ stretch mode (r^+^), the CH_2_ Fermi resonance (d^+^_FR_), the asymmetric (as-) CH_2_ stretch mode (d^–^), the CH_3_ Fermi resonance (r^+^_FR_), and the as-CH_3_ stretch (r^–^) respectively. The amplitude ratio between the s-CH_2_ mode and the s-CH_3_ mode (d^+^/r^+^ ratio) is correlated to the alkyl chain conformation of the surfactant molecules at the interface. A d^+^/r^+^ ≪ 1 ratio means that alkyl chains are stretched in an all-trans conformation, whereas a d^+^/r^+^ > 1 ratio corresponds to the presence of gauche defects in the surfactant monolayer.^37–39^ The s-CH_2_ peak dominates, with a resulting d^+^/r^+^ ratio of ∼14.5 (Supporting Information, Table S3) for the oil-in-water droplets. This means that the Span80 alkyl chains form disordered structures at the surface of oil droplets in water. By contrast, the vibrational SFS spectrum of the water-in-oil droplet interface (Figure 2C, blue data) contains s-CH_2_ and s-CH_3_ peaks with a d^+^/r^+^ amplitude ratio of ∼1.2. This lower ratio indicates that Span80 molecules acquire a significantly more ordered conformation with fewer gauche defects at the water droplets in oil interface, compared to that of the oil droplets in water interface, in agreement with a previous investigation.^40^ Thus, even though the interface is composed of the same chemicals, Span80 adopts different configurations, which depend on whether the bulk phase is composed of oil or water. The structural differences are schematically illustrated in the inset of Figure 2C. SFS spectra of a similar droplet system were published earlier in ref 40 as part of a study on spectroscopic interference and adsorption effects. Both sets of spectra are very similar.

The difference in the surface structure of Span80 at the two droplet interfaces is likely related to their different interfacial droplet geometries and stabilizing mechanisms. The Span80 covered hexadecane oil droplets have a negative ζ-potential value (−46 mV) that is a bit smaller in magnitude than that of bare hexadecane droplets in water (−56 mV, ref 25). Span80 is a neutral surfactant, and therefore does not contribute to the net charge on the droplet when it is adsorbed at the interface. Meanwhile, Span80 is sparingly soluble in water, and thus partitions favorably into the oil phase. Due to the convex geometry of the oil droplet surface, Span80 molecules are present in the interfacial region with highly disordered alkyl chains, leading to incompletely covered oil–water interfaces (which was also concluded recently in an interference study^40^). For pure oil droplets in water, the negative electrophoretic mobility stems from improper H-bonds between oil C–H (charge accepting) and water O–H (charge donating) groups,^25,41^ which provide a weak negative surface charge. pH-dependent all-optical surface potential and SFS measurements in combination with ab initio molecular dynamics simulations further showed that the negative charge at the interface is pH-independent, and most likely arises from charge transfer from the water to the oil phase.^20^ Charge transfer between water and interfacial oil thus ensures that neat oil droplets in water are weakly negatively charged. Since Span80-coated oil droplets are only partially covered with Span80 and also possess bare oil–water contacts,^40^ the mechanism that stabilizes the bare oil droplets and the Span80 covered oil droplets is probably similar. The stabilizing mechanism for Span80 covered oil droplets is therefore mostly electrostatic in nature, and the negative charge which repels the droplets from each other arises from charge transfer interactions.^25^ In addition, there is a potential steric contribution, which is due to repulsion between the alkyl chains of the Span80 that partially protrude into the aqueous phase, as illustrated in Figure 2C.

Water droplets in oil likely have a different balance of interactions, which is already indicated by the different electrostatics, and the much lower electrophoretic mobility value (μ ∼ −2 vs 0.01 × 10^–8^ m^2^/(V s)). Since the alkyl chains from Span80 stick out into the main oil phase with a concave geometry, enabling more straight alkyl chains, the steric repulsion mechanism should be more pronounced. To investigate the interfacial structure in more detail, we next consider the interfacial vibrational response of water. The interfacial vibrational response of water arises from second-order interfacial interactions as well as from the effective third-order electric field-induced interactions in the double layer region.^27,42^

### Interfacial Water: Finite Volume Effects

Figure 3A shows the interfacial water spectra from the O–D stretch, |Γ^(2)^|^2^, of bare oil nanodroplets in water (red data), together with Span80 covered oil droplets in water (black data) and the air–water interface (green data). Figure 3B shows the vibrational O–D stretch spectra for the two inverted nanodroplet interfaces, oil droplets in water with Span80 (black, the same spectrum as in Figure 3A) and water droplets in oil with Span80 (blue data). The concentration of Span80 is 10 mM in both oil phases.

The intensity between 2200 and 2800 cm^–1^ originates from the O–D stretch modes of interfacial D_2_O molecules. These interfacial vibrational modes include O–D stretches from water molecules with an anisotropic molecular orientation with respect to the surface normal. The anisotropy originates either from the interaction with the weak interfacial electrostatic field that arises from the charge on the oil droplets, or from chemical interactions due to surface chemistry, such as H-bonding.^42^ The Span80 covered oil-droplet-in-water spectrum (Figure 3A, black data) is similar to previously published bare oil-droplet-in-water SF spectrum (reproduced in Figure 3A, red data),^25,43^ having a broad spectral shape with 3 distinct features, indicated by the dashed lines at ∼2390 and ∼2510 cm^–1^, and one at ∼2630 cm^–1^. The first two and their ratio are indicative of the strength of the H-bond network as the 2390/2510 ratio increases at lower temperatures. With a spectral shape that has greater lower frequency intensities compared to the air–water (green data) and bare oil-droplet water interfaces, the H-bond network around the Span80-oil-droplet-water interface is more strongly ordered. The third (weaker) feature at ∼2630 cm^–1^ arises from water molecules that are not H-bonded to other water molecules, but instead are H-bonded to the C–H groups of the oil interface.^25^ These are very weak H-bonds, called improper H-bonds, with a strength on the order of the thermal energy (kT), that are responsible for charge transfer between the oil and the water, resulting in a net negative charge on the oil droplets.^20,25^ Spectrally, this leads to a peak having the same symmetry as the free O–D mode at the air–water interface (at 2745 cm^–1^ in Figure 3A) but broadened and red-shifted by ∼100 cm^–1^. Because the Span80 covered oil droplets in water and the bare oil droplets in water have similar vibrational water spectra and comparable electrophoretic mobilities, the overall interactions (charge transfer and H-bonding) seem to be very similar. As both samples have very low ionic strength (∼1 μM), the spectra are dominated by both second-order effects (such as the improper H-bonds) as well as third-order effects (the interaction between the interfacial electrostatic field and the water molecules inside the diffuse electric double layer).

Next, we compare the interfacial water structure of incompletely Span80 covered oil-droplets-in-water (Figure 3A,B, black data) to water-droplets-in-oil (Figure 3B, blue data). The spectra are quite different. Both interfacial water spectra contain features around 2390/2510 and 2630 cm^–1^, but with notable differences. Specifically, the red side of the water droplet spectrum has a higher intensity and a lower frequency (2370 cm^–1^ for the maximum intensity frequency), while the 2510 cm^–1^ feature shows the opposite trend in terms of relative intensity. Also, the ‘dip’ in intensity around 2460 cm^–1^ that is visible in the black interfacial water spectrum next to the oil droplet is not present in the blue interfacial water spectrum of the water droplet. The higher intensity and lower frequency (2370 cm^–1^) of the water droplet spectrum suggests that interfacial water from the water droplet has a higher population of more strongly H-bonded water molecules. It should be noted that water molecules inside water droplets are subject to the opposite strain as those outside oil droplets (Figure 3C) due to their inversed geometries (convex for oil droplets vs concave for water droplets). Figure 1 shows that, among the three geometries compared, with identical surface charge, ionic strength and dielectric constants, oil droplets (Figure 1B,E,H) in water have the strongest electrostatic interfacial field, whereas water droplets (Figure 1C,F,I) have an internal electrostatic field that is virtually absent.

The spectral differences could be explained by the presence of the surfactant, or by finite volume effects (or a combination of both). In terms of the surfactant, Figure 3A already showed that Span80 does not have a major impact on the water structure. For the case of water droplets in hexadecane oil, which are not stable without the surfactant, SF spectra of the aqueous interface were recorded with water droplets stabilized by other nonionic surfactants (see ref 36). These spectra are quite similar. Here, we further examine the influence of the surfactant by replacing the nonionic surfactant with an anionic surfactant (AOT, aerosol OT, sodium bis­(2-ethylhexyl) sulfosuccinate, with the chemical structure given in Figure 3D inset) at 1 mM concentration in CCl_4_. Figure 3D shows the comparison of SF water spectra between water droplets prepared with Span80 in d-C16 (blue data in Figure 2B) and AOT in CCl_4_ (purple data). Despite the use of a different bulk phase, CCl_4_, and a different surfactant, AOT, with distinct chemical structures, the recorded spectrum from water droplets remains similar to those formed by Span80 in d-C16. Therefore, the surfactant–water interactions are not likely the determining factor for water structure in water droplets or its spectral changes from oil droplets seen in Figure 3B. We note that observations pertaining to nonionic surfactant structure and their influence on the SF spectrum of interfacial water are different from the planar air–water interface, where generally much bigger effects are observed, often leading to the suppression of the interfacial water response.^44–46^

Finite volume effects can have a major impact on the electrostatic field, as the electrostatic potential computations in Figure 1 showed. For water droplets, an almost constant electrostatic field of negligible amplitude is present throughout the entire water droplet. The field lines initially converge and then swerve in order to avoid singularities^19^ (Figure 1C, indicated by the dashed lines). For the oil-in-water system, an electrostatic field exists, which is larger in magnitude than that inside the water droplet and outside the planar oil interface. This field decays to 0 in the direction of the surface normal (Figure 1B). Another finite volume effect pertains to the ratio of water to oil molecules and the fact that there are progressively fewer water molecules in each ‘layer’ as moving away from the water droplet interface into the center. Finally, a difference between both systems is the curvature/geometry of the interface which potentially influences the Span80 conformation, and can also impact the interfacial water.^16,47,48^ However, as the surfactant does not drastically impact the water structure as evidenced by the SFS experiment, this effect is probably not of primary relevance.

Because the broad shape of both water spectra in Figure 3B are also potentially determined by intermolecular and intramolecular vibrational coupling,^49–52^ further experiments were undertaken to disentangle these effects from the finite volume effects mentioned above. Therefore, to better understand the structure of water, we next decouple finite volume effects from vibrational coupling by isotope dilution to reveal the characteristics of the H-bonding network around oil droplets and inside water droplets, both covered with the same amounts of Span80 as in Figures 2C and 3B.

### Decoupling Inter- and Intramolecular Vibrational Modes

In addition to the mentioned finite volume effects in Figure 3B (a difference in electrostatics, the local molecular environment and H-bonding), the spectra are likely also influenced by intra- and intermolecular coupling.^51^ The vibrations of water molecules are not isolated but rather distributed among several molecules and vibrational modes. Auer and Skinner^49^ estimated in a computational study that each O–D vibrational mode is linked to up to 12 neighboring O–D oscillators that have similar vibrational energies. This intermolecular coupling leads to a broadening of the O–D stretch spectrum, as is illustrated in Figure 4A.^49–51^ Additionally, the vibrational energy of water molecules can delocalize over different vibrational modes of the same molecule via intramolecular coupling (Figure 4B): As the O–D stretch modes and the overtone of D–O–D bending modes have similar vibrational energies, they are coupled with each other via a Fermi resonance. Intramolecular coupling leads to energy level splitting and peak broadening of the O–D stretch vibrations.^52^ It also results in the appearance of a spectral intensity minimum or dip, called an Evans window.^53^

To decouple local H-bonding environment (i.e., the presence of more strongly/weakly H-bonded regions and their distribution) and vibrational coupling effects, isotopic dilution experiments were performed for both types of droplet systems. The same droplets systems were used as in Figures 2C and 3B, but now with isotope dilution of the aqueous phase. In order to disentangle the O–D structural differences from intra- and intermolecular vibrational coupling, we used a mole ratio of D_2_O of 15% in H_2_O and probed the O–D oscillators. In this way, coupling effects, such as spectral broadening and splitting, were minimized since the energy levels of the probed vibrational O–D modes were different from those that surround them (mainly O–H groups). Note that it has been concluded that isotope dilution does not disturb the configuration of the H-bond network itself, insofar as it is probed by the OD/OH stretch vibrational modes.^52^

Figure 4C displays the O–D stretch spectra of the incompletely Span80 covered interfaces of oil-in-water droplets. The water phase was isotopically diluted with D_2_O:H_2_O volume ratios of 100:0, 85:15, 50:50, and 15:85, leading to mole fraction mixtures of (D_2_O: HOD:H_2_O) of (1,0,0), (0.73:0.25:0.02), (0.25:0.5:0.25), and (0.02:0.25:0.73) respectively. The 15:85 mole fraction mixture almost entirely reflects the uncoupled O–D stretch modes, as it contains 25% vibrationally uncoupled HOD and just 2% D_2_O. Note that the OH stretch modes are not probed. Figure 4C shows that, upon isotope dilution, the feature at 2390 cm^–1^ is strongly reduced and the spectrum narrows to an almost symmetric single peak centered at ∼2506 cm^–1^, with a full-width-at-half-maximum (fwhm) of 140 cm^–1^ (see Section S3 for details). This means that the spectral shape of water is significantly influenced by vibrational coupling, including intermolecular and intramolecular coupling. The intramolecular coupling is comprised of an interaction between the overtone of the O–D bend mode with the O–D stretch mode (Figure 4B). The intensity ‘minimum’ between the two features at ∼2390 and 2510 cm^–1^ is the so-called Evans window.^53^ This behavior is very similar to what was previously recorded for bare oil droplets in water (see ref 43, also for an in-depth comparison to the air–water interface). On the high frequency side of Figure 4C, up to 2700 cm^–1^, there is still SF intensity at every dilution fraction indicating that there are still water molecules that are not H-bonded to other water molecules, which can participate in charge transfer with the interfacial oil molecules. Note that the oil droplet behavior is different from the air–water interface, where no high frequency component remains.^54^

Next, we examined the behavior of the inverse system, water droplets in oil. The O–D stretch spectrum of the interfaces of 100% D_2_O droplets that are incompletely covered with Span80 has a low frequency O–D stretch feature around 2370 cm^–1^, and a fwhm of 194 cm^–1^ (see Section S3 for details). Increasing the amount of H_2_O in the aqueous phase, the O–D stretch mode becomes uncoupled, and shifts to higher frequencies, maximizing at 2506 cm^–1^, the same center frequency as for the oil droplets-in-water system, with a fwhm of 212 cm^–1^ (see Section S3 for details). As such, upon decoupling, the spectrum shifts to higher frequencies with the spectral width remaining practically unchanged. Figure 4E shows the HOD spectra for both droplet systems (with 15% D_2_O) plotted together. Figure 4F shows the change in fwhm as a function of D_2_O fraction for both systems. The error bars (shaded curves) indicate the uncertainty that is mainly due to an elevated baseline (induced by the IR absorption of the deuterated hexadecane) and the spectral line shape, which is not symmetric. For oil droplets in water, vibrational decoupling reduces the spectral width by ∼50%, while for water droplets in oil, no width reduction is seen within experimental error.

There is thus a clear difference in frequency distributions for the HOD spectra of oil droplets in water and water droplets in oil, made using the same chemicals, with comparable droplet sizes and concentrations. Vibrationally uncoupled water molecules inside water droplets exhibit spectral intensities at both higher and lower frequencies than the inverse oil droplet system. This means that the interfacial environments are quite different with the water droplet being more environmentally heterogeneous. Water droplets have a relatively higher population of water molecules experiencing higher degrees of ordering, and also a higher population of water molecules experiencing lower degrees of ordering. In other words, there are water molecules at the water droplet interface that are more strongly and more weakly H-bonded compared to water molecules at the oil-droplet or air–water interface. For water droplets, the uncoupled O–D stretch mode is markedly broader compared to the oil-in-water droplet surface and bulk water. This difference likely comes from the concave vs convex geometries as shown in Figure 3C. There are approximately one million water molecules on the surface of a 100 nm droplet. However, inside water droplets, the number of water molecules decreases significantly in each subsequent layer, corresponding to a greater percentage mismatch between neighboring layers, as one moves toward the center of the droplet. In this case, the water layers become progressively further out of registry with the previous layer toward the middle of the droplet. As a consequence, there will be more broken hydrogen bonds, and the water spectrum thus shows more heterogeneous line broadening.

Furthermore, no change in the spectral width is observed upon isotope dilution (Figure 4F), which means that vibrational coupling is not playing a decisive role in water droplets. This is also apparent from the spectral shape of 100% D_2_O droplets, which is asymmetric and does not display an Evans window. The reduction of vibrational coupling is likely due to the change in the interfacial environment that creates a more heterogeneous H-bonding environment in the interfacial region. This results in a broader vibrational spectrum for the HO–D stretch modes. Since the H-bond environment impacts the O–D bending mode to a lesser extent,^55–57^ the intramolecular coupling might be greatly diminished and thus results in different spectral shapes. In this case, the intermolecular vibrational coupling dominates as demonstrated by the shift in the spectrum to higher frequency upon isotope dilution.

Thus, there is a striking difference between the two inverse systems upon isotope dilution (vibrational uncoupling). Namely, the SFS spectrum of water at the oil-droplet-in-water interface transforms from double-featured broad resonances with a fwhm of ∼255 cm^–1^ to a nearly single-featured symmetric band with a fwhm of ∼140 cm^–1^. The low-frequency part loses intensity while the high-frequency part remains very similar. Vibrational coupling thus results in an increase of lower frequencies as well as spectral broadening. This behavior originates primarily from intramolecular vibrational coupling. It is similar in behavior to what has been seen on the pure oil-droplet-in-water and air–water interfaces.^43^ Interestingly, the SFS spectrum of the water-droplet-in-oil system behaves very differently. Upon isotope dilution, the O–D spectrum changes with the low-frequency modes losing intensity, while the high-frequency modes gain intensity. Since the spectral width remains the same, the intramolecular vibrational coupling mechanism appears to be less prominent than the intermolecular coupling one. This results in a frequency shift of the whole spectrum. Comparing the vibrationally decoupled oil-droplets-in-water to water-droplets-in-oil, there are both more strongly and more weakly H-bonded configurations inside the water-droplets’ interfacial region. Also, the water has larger orientational anisotropy. These differences can potentially be attributed to three finite-volume effects, which were already discussed partially in the context of Figure 3:

(1) Convergent vs divergent electric fields: the two inverse droplet systems exhibit distinct surface geometries (Figure 3C), which give rise to a strong divergent electric field from oil droplets into the bulk, but a weak convergent field from the water droplets surface into the center (Figure 1). For a charged planar interface, the field next to it attenuates parallel to the surface normal (Figure 1D). While all systems have ionic and dielectric screening, the water droplets have a much weaker potential decay (Figure 1F). By comparison, the electrostatic potential around oil droplets drops much faster as a function of distance, because of its divergent geometry, which leads to a thinner double layer that has a relatively stronger electrostatic field (Figure 1E).In contrast, a water droplet submerged in a nonconductive oil phase has close-to-zero electrophoretic mobility, and at (sub) micromolar ionic strength has a nearly absent electrostatic field throughout the entire droplet (including the interface). As a consequence, the contribution from third-order electrostatic field interactions to the SFS spectrum should be largest for the oil droplet in water system, and somewhat smaller for a planar oil–water interface. Since there is practically no electrostatic field in the water droplets, third-order effects do not contribute to the water droplet SFS spectrum.
(2) Opposite strains upon water molecules: the water molecules around oil droplets and inside water droplets have opposite strains under the imposed surface geometries (Figure 3C). The H-bond network is formed between neighboring water molecules across different layers next to the interfaces. As one moves away from oil droplet surfaces, the available space and number of water molecules needed for forming subsequent layers gradually increase due to their convex geometry. In this case, the H-bonding structure is potentially not strongly perturbed because the relative difference in the number of water molecules in each subsequent layer is rather small. As such, the water molecules could remain in registry. However, under concave geometry, the number of water molecules in each subsequent layer decreases sharply as the center of water droplets is approached. This geometrical restriction results in out-of-registry water layers, which are likely to have more broken H-bonds. Hence, there is a more heterogeneous water environment and a much broader HOD spectrum (Figure 4E) inside water droplets than outside oil droplets.
(3) Finite volume impacts the formation of the electric double layer: at an interface in contact with an infinitely large, ideal, bulk aqueous phase, the distribution of ions is driven by local ion-surface, ion–ion, ion–water interactions, but also by nonlocal entropic effects. A finite volume effect is expected to influence the balance of interactions and in particular the entropic contribution. Further investigations involving experiments more targeted at understanding electrostatic interactions inside water droplets are needed to fully understand this mechanism.

## Conclusions

We investigated the structure of water surrounding oil nanodroplets and water nanodroplets embedded in oil prepared using the same three chemicals (hexadecane, Span80, and water), with the aim to understand finite volume effects on the molecular level. Using vibrational sum frequency scattering, we investigated the interfacial structure of water and surfactant molecules. Water droplets display a much smaller net electrophoretic mobility than oil droplets. Span80 incompletely covers both interfaces having somewhat more disordered interfacial structures for oil nanodroplets-in-water versus water nanodroplets-in-oil. The interfacial water structure around Span80 carrying oil droplets had a similar shape to the water spectrum around bare oil droplets. The water spectrum of the interface of water droplets in oil did not vary in shape when Span80 was exchanged for another surfactant. Both observations support the notion that finite volume effects are the main drivers behind spectral changes. Comparing the water structure at the interface of oil-in-water droplets to water-in-oil droplets, we find that interfacial water molecules exhibit drastically different structures. Specifically, interfacial water molecules inside the water-in-oil droplets are more strongly hydrogen-bonded compared to interfacial water molecules outside oil-in-water droplets. We further investigated the interfacial structures using isotopic dilution, which was used to generate a sum frequency spectrum that is not influenced by intra- or intermolecular coupling.

The uncoupled O–D spectrum of water-in-oil droplets is broader than that of the oil-in-water droplets, implying the presence of a more structured liquid that also has a broader range of H-bonding strengths at this interface. The presence of both more strongly hydrogen bonded and more weakly hydrogen bonded water molecules compared to the oil-in-water droplet surface reveals that the water-in-oil droplet surface is more heterogeneous in nature than the oil droplet in water interface. The vibrational coupling mechanism is also different. For oil droplets in water, the SFS spectrum is dominated by intramolecular coupling, while for water nanodroplets, the SFS spectrum is dominated by intermolecular coupling.

## Methods

### Chemicals

Hexadecane (C_16_H_34_, C16, 99.8%, Sigma-Aldrich), d_34_-hexadecane (98% D, Cambridge Isotope), D_2_O (99.8% D, Thermo Scientific) with an electrical resistance >2 MΩ·cm (conductivity 0.5 μS/cm), Span80 (sorbitanemonooleate, Sigma-Aldrich) were used as received. The purity of hexadecane was verified with a Zisman test.^58^ H_2_O used was ultrapure water (Milli-Q UF plus, Millipore, Inc.) with an electrical resistance 18.2 MΩ·cm (conductivity 0.055 μS/cm). The glassware used for preparing and storing nanoemulsions was freshly taken out of the manufacturer’s packaging and never reused after the preparation. As a first step, the glassware was cleaned with a freshly prepared piranha (3:1 H_2_SO_4_:H_2_O_2_) solution. After ∼20 min immersion, the glassware was rinsed copiously with ultrapure water.

### Sample Preparation and Characterization

The stock nanodroplets were prepared using a two-step process. First, 2 vol % droplet material (oil or water) was mixed with 98 vol % the main phase (water or oil) in a 4 mL glass vial. The oil phase contained 10 mM Span80. The water phase was prepared with either 100% D_2_O (Figure 2 and 3) or containing different D_2_O:H_2_O ratios (Figure 4). For 1 vol % oil droplets in D_2_O:H_2_O were obtained from the dilution of the stock emulsion. The mixture was homogenized using a hand-held homogenizer (TH, OMNI International) at an angular velocity of 15 rpm for 3–5 min. Nanoemulsions were obtained by consecutive sonication in an ultrasonic bath (35 kHz, 400 W, Bandelin) for 3–10 min. The size distribution of droplets was characterized by dynamic light scattering (DLS) using a Malvern ZS nanosizer instrument and had average diameters varying between ∼100–300 nm (see Section S1 and Table S1). The electrophoretic mobilities of different emulsions (see Section S1 and Table S2) were measured using the same Malvern ZS nanosizer instrument. More details on the preparation procedure, including the effect of different cleaning procedures on the prepared nanoemulsions are discussed in detail in ref 41.

### Vibrational SFS Spectroscopy Setup

The experimental setup for the vibrational SFS spectroscopy has been described in detail previously.^22^ Briefly, an 800 nm regeneratively amplified Ti:sapphire system (Spitfire Pro, Spectra physics) seeded with an 80 MHz 800 nm oscillator (MaiTai SP) was operated at a 1 kHz repetition rate to pump a commercial OPG/OPA/DFG system (HE-TOPAS-C, Light Conversion) to generate infrared (IR) pulses. The visible beam was split off directly from the amplifier and spectrally shaped with a home-built pulse shaper. The visible (800 nm, 10 μJ, fwhm 15 cm^–1^) and the IR (3–4.5 μm, 10 μJ, fwhm 170 cm^–1^) beams were spatially and temporally overlapped inside a 200 μm sample cell with an IR-VIS opening angle of 15°. At a scattering angle (θ, measured in air) of 57°, the scattered SF light was collimated using a plano-convex lens (*f* = 15 mm, Thorlabs LA1540-B) and passed through two short-wave pass filters (third Millenium, 3RD770SP). The SF light was spectrally dispersed with a monochromator (Acton, SpectraPro 2300i) and detected with an intensified CCD camera (Princeton Instruments, PI-Max3) using a gate width of 10 ns. The acquisition time for a single spectrum was 600 s. A Glan-Taylor prism (Thorlabs, GT15-B), a half-wave plate (EKSMA, 460-4215) and a polarizing beam splitter cube (CVI, PBS-800-050) and two BaF_2_ wire grid polarizers (Thorlabs, WP25H-B) were used to control the polarization of the SF, VIS and infrared beams, respectively. The SFS spectra that are reported in this work were recorded using P-polarized (parallel to the horizontal scattering plane) IR, and S polarized (perpendicular to the horizontal scattering plane) SF and VIS beams respectively, abbreviated as the SSP polarization combination.

## Supplementary Material



## Notes

Electrokinetic mobility measurements and SFS spectra acquisition (S1); method of retrieving |Γ^(2)^|^2^ spectrum from measured SF intensity (S2); SFS spectra with error bar and spectral bandwidth calculation (S3) (PDF)

## References

[1] Köhler M. H., Bordin J. R., de Matos C. F., Barbosa M. C. (2019). Water in Nanotubes:
The Surface Effect. Chem. Eng. Sci..

[2] Knight A. W., Kalugin N. G., Coker E., Ilgen A. G. (2019). Water Properties
under Nano-Scale Confinement. Sci. Rep..

[3] Gao Z., Giovambattista N., Sahin O. (2018). Phase Diagram of Water
Confined by
Graphene. Sci. Rep..

[4] Cicero G., Grossman J. C., Schwegler E., Gygi F., Galli G. (2008). Water Confined
in Nanotubes and between Graphene Sheets: A First Principle Study. J. Am. Chem. Soc..

[5] Hummer G., Rasaiah J. C., Noworyta J. P. (2001). Water Conduction
Through the Hydrophobic
Channel of a Carbon Nanotube. Nature.

[6] Bocquet L., Charlaix E. (2010). Nanofluidics, from
Bulk to Interfaces. Chem. Soc. Rev..

[7] Chakraborty S., Kumar H., Dasgupta C., Maiti P. K. (2017). Confined Water:
Structure, Dynamics, and Thermodynamics. Acc.
Chem. Res..

[8] Hassan J., Diamantopoulos G., Homouz D., Papavassiliou G. (2016). Water inside
Carbon Nanotubes: Structure and Dynamics. Nanotechnol.
Rev..

[9] Pascal T. A., Goddard W. A., Jung Y. (2011). Entropy and
the Driving Force for
the Filling of Carbon Nanotubes with Water. P. Natl. Acad. Sci. USA.

[10] Tsimpanogiannis I. N., Moultos O. A., Franco L. F. M., Spera M. B. D., Erdos M., Economou I. G. (2019). Self-Diffusion Coefficient
of Bulk and Confined Water:
a Critical Review of Classical Molecular Simulation Studies. Mol. Simul..

[11] Anovitz L. M., Cole D. R. (2015). Characterization
and Analysis of Porosity and Pore
Structures. Rev. Mineral. Geochem..

[12] Klameth F., Vogel M. (2013). Structure and Dynamics of Supercooled Water in Neutral Confinements. J. Chem. Phys..

[13] Mondal S., Bagchi B. (2024). Dielectric Properties of Nanoconfined Water. J. Chem. Phys..

[14] Kumar S., Bagchi B. (2022). Correlation Lengths in Nanoconfined Water and Transport
Properties. J. Chem. Phys..

[15] Yamada S. A., Hung S. T., Thompson W. H., Fayer M. D. (2020). Effects of Pore
Size on Water Dynamics in Mesoporous Silica. J. Chem. Phys..

[16] Chandler D. (2005). Interfaces
and the Driving Force of Hydrophobic Assembly. Nature.

[17] Hunter, R. J. Zeta Potential in Colloid Science: Principles and Applications; Academic Press, 1981.

[18] Ohshima H. (2021). Electrophoretic
Mobility of a Liquid Drop with a Slip Surface. Colloid Polym. Sci..

[19] Uematsu Y., Ohshima H. (2022). Electrophoretic Mobility
of a Water-in-Oil Droplet
Separately Affected by the Net Charge and Surface Charge Density. Langmuir.

[20] Pullanchery S., Kulik S., Schönfeldová T., Egan C. K., Cassone G., Hassanali A., Roke S. (2024). pH Drives Electron
Density Fluctuations that Enhance Electric Field-Induced Liquid Flow. Nat. Commun..

[21] De
Aguiar H. B., De Beer A. G. F., Strader M. L., Roke S. (2010). The Interfacial
Tension of Nanoscopic Oil Droplets in Water Is Hardly Affected by
SDS Surfactant. J. Am. Chem. Soc..

[22] De
Aguiar H. B., Strader M. L., De Beer A. G. F., Roke S. (2011). Surface Structure
of Sodium Dodecyl Sulfate Surfactant and Oil at the Oil-in-Water Droplet
Liquid/Liquid Interface: A Manifestation of a Nonequilibrium Surface
State. J. Phys. Chem. B.

[23] Zdrali E., Chen Y., Okur H. I., Wilkins D. M., Roke S. (2017). The Molecular
Mechanism of Nanodroplet Stability. ACS Nano.

[24] Zdrali E., Etienne G., Smolentsev N., Amstad E., Roke S. (2019). The Interfacial
Structure of Nano- and Micron-Sized Oil and Water Droplets Stabilized
with SDS and Span80. J. Chem. Phys..

[25] Pullanchery S., Kulik S., Rehl B., Hassanali A., Roke S. (2021). Charge Transfer Across C-H···O Hydrogen Bonds Stabilizes
Oil Droplets in Water. Science.

[26] de
Beer A. G. F., Roke S. (2010). Obtaining Molecular Orientation from
Second Harmonic and Sum Frequency Scattering Experiments in Water:
Angular Distribution and Polarization Dependence. J. Chem. Phys..

[27] Gonella G., Lütgebaucks C., de Beer A. G. F., Roke S. (2016). Second Harmonic and
Sum-Frequency Generation from Aqueous Interfaces is Modulated by Interference. J. Phys. Chem. C.

[28] de
Beer A. G. F., Roke S. (2016). What Interactions Can Distort the
Orientational Distribution of Interfacial Water Molecules as Probed
by Second Harmonic and Sum Frequency Generation?. J. Chem. Phys..

[29] van
der Loop T. H., Ottosson N., Lotze S., Kentzinger E., Vad T., Sager W. F. C., Bakker H. J., Woutersen S. (2014). Structure
and Dynamics of Water in Nanoscopic Spheres and Tubes. J. Chem. Phys..

[30] Groot C. C. M., Velikov K. P., Bakker H. J. (2016). Structure and Dynamics of Water Molecules
Confined in Triglyceride Oils. Phys. Chem. Chem.
Phys..

[31] Fayer M. D., Levinger N. E. (2010). Analysis of Water in Confined Geometries and at Interfaces. Annu. Rev. Anal. Chem..

[32] Pieniazek P. A., Lin Y. S., Chowdhary J., Ladanyi B. M., Skinner J. L. (2009). Vibrational
Spectroscopy and Dynamics of Water Confined inside Reverse Micelles. J. Phys. Chem. B.

[33] Pullanchery S., Dupertuis N., Roesel T., Roke S. (2023). Liposomes and Lipid
Droplets Display a Reversal of Charge-Induced Hydration Asymmetry. Nano Lett..

[34] Strazdaite S., Versluis J., Backus E. H. G., Bakker H. J. (2014). Enhanced Ordering
of Water at Hydrophobic Surfaces. J. Chem. Phys..

[35] Strazdaite S., Versluis J., Bakker H. J. (2015). Water Orientation
at Hydrophobic
Interfaces. J. Chem. Phys..

[36] Smolentsev N., Smit W. J., Bakker H. J., Roke S. (2017). The Interfacial Structure
of Water Droplets in a Hydrophobic Liquid. Nat.
Commun..

[37] Esenturk O., Walker R. A. (2006). Surface Vibrational
Structure at Alkane Liquid/Vapor
Interfaces. J. Chem. Phys..

[38] Guyotsionnest P., Hunt J. H., Shen Y. R. (1987). Sum-Frequency Vibrational Spectroscopy
of a Langmuir Film - Study of Molecular-Orientation of a Two-Dimensional
System. Phys. Rev. Lett..

[39] Tyrode E., Hedberg J. (2012). A Comparative Study
of the CD and CH Stretching Spectral
Regions of Typical Surfactants Systems Using VSFS: Orientation Analysis
of the Terminal CH and CD Groups. J. Phys. Chem.
C.

[40] Pullanchery S., Zhang L., Kulik S., Roke S. (2023). Interfacial Inversion,
Interference, and IR Absorption in Vibrational Sum Frequency Scattering
Experiments. J. Phys. Chem. B.

[41] Pullanchery S., Kulik S., Okur H. I., de Aguiar H. B., Roke S. (2020). On the Stability and Necessary Electrophoretic Mobility of Bare Oil
Nanodroplets in Water. J. Chem. Phys..

[42] de
Beer A. G. F., Campen R. K., Roke S. (2010). Separating Surface
Structure and Surface Charge with Second-Harmonic and Sum-Frequency
Scattering. Phys. Rev. B.

[43] Pullanchery S., Kulik S., Roke S. (2022). Water Structure
at the Hydrophobic
Nanodroplet Surface Revealed by Vibrational Sum Frequency Scattering
Using Isotopic Dilution. J. Phys. Chem. B.

[44] Tyrode E., Johnson C. M., Kumpulainen A., Rutland M. W., Claesson P. M. (2005). Hydration
State of Nonionic Surfactant Monolayers at the Liquid/Vapor Interface:
Structure Determination by Vibrational Sum Frequency Spectroscopy. J. Am. Chem. Soc..

[45] Tyrode E., Johnson C. M., Rutland M. W., Claesson P. M. (2007). Structure and Hydration
of Poly (Ethylene Oxide) Surfactants at the Air/Liquid Interface.
A Vibrational Sum Frequency Spectroscopy Study. J. Phys. Chem. C.

[46] Kusaka R., Ishiyama T., Nihonyanagi S., Morita A., Tahara T. (2018). Structure
at the Air/Water Interface in the Presence of Phenol: a Study using
Heterodyne-Detected Vibrational Sum Frequency Generation and Molecular
Dynamics Simulation. Phys. Chem. Chem. Phys..

[47] de
la Puente M., Laage D. (2024). Impact of Interfacial Curvature on
Molecular Properties of Aqueous Interfaces. J. Chem. Phys..

[48] Stiopkin I. V., Weeraman C., Pieniazek P. A., Shalhout F. Y., Skinner J. L., Benderskii A. V. (2011). Hydrogen Bonding at the Water Surface Revealed by Isotopic
Dilution Spectroscopy. Nature.

[49] Auer B. M., Skinner J. L. (2008). IR and Raman Spectra of Liquid Water: Theory and Interpretation. J. Chem. Phys..

[50] Woutersen S., Bakker H. J. (1999). Resonant Intermolecular Transfer of Vibrational Energy
in Liquid Water. Nature.

[51] Ramasesha K., De Marco L., Mandal A., Tokmakoff A. (2013). Water Vibrations
have Strongly Mixed Intra- and Intermolecular Character. Nat. Chem..

[52] Sovago M., Campen R. K., Wurpel G. W. H., Müller M., Bakker H. J., Bonn M. (2008). Vibrational
Response of Hydrogen-Bonded
Interfacial Water is Dominated by Intramolecular Coupling. Phys. Rev. Lett..

[53] Evans J. C., Wright N. (1960). A Peculiar Effect in
the Infrared Spectra of Certain
Molecules. Spectrochim. Acta.

[54] Sovago M., Campen R. K., Bakker H. J., Bonn M. (2009). Hydrogen Bonding Strength
of Interfacial Water Determined with Surface Sum-Frequency Generation. Chem. Phys. Lett..

[55] Yu C.-C., Chiang K.-Y., Okuno M., Seki T., Ohto T., Yu X., Korepanov V., Hamaguchi H., Bonn M., Hunger J., Nagata Y. (2020). Vibrational
Couplings and Energy Transfer Pathways
of Water’s Bending Mode. Nat. Commun..

[56] Seki T., Sun S. M., Zhong K., Yu C. C., Machel K., Dreier L. B., Backus E. H. G., Bonn M., Nagata Y. (2019). Unveiling
Heterogeneity of Interfacial Water through the Water Bending Mode. J. Phys. Chem. Lett..

[57] Seki T., Chiang K. Y., Yu C. C., Yu X. Q., Okuno M., Hunger J., Nagata Y., Bonn M. (2020). The Bending Mode of
Water: A Powerful Probe for Hydrogen Bond Structure of Aqueous Systems. J. Phys. Chem. Lett..

[58] Bigelow W. C., Pickett D. L., Zisman W. A. (1946). Oleophobic
Monolayers 0.1. Films
Adsorbed from Solution in Non-Polar Liquids. J. Col. Sci..

