# Supplementary material for: Finite Volume Effects in Water Nanodroplets: A Molecular Level Investigation
Source: ACS Nano. 2025 Jun 24;19(26):23829–39. doi: 10.1021/acsnano.5c04422 (PMC12257642; doi:10.1021/acsnano.5c04422)
Supplement: Supplementary file 1 [file nn5c04422_si_001.pdf]

Supporting Information for:

Finite volume effects in water nanodroplets: A molecular level investigation

Li Zhang[a], Saranya Pullanchery[a], Paul S. Cremer[d*], and Sylvie Roke[a,b,c*]

[a]Laboratory for fundamental BioPhotonics, Institute of Bioengineering (IBI), School of Engineering (STI), École Polytechnique Fédérale de Lausanne (EPFL), CH-1015 Lausanne, Switzerland.

[b]Institute of Materials Science and Engineering (IMX), School of Engineering (STI), École Polytechnique Fédérale de Lausanne (EPFL), CH-1015 Lausanne, Switzerland.

[c]Lausanne Centre for Ultrafast Science, École Polytechnique Fédérale de Lausanne (EPFL), CH-1015 Lausanne, Switzerland.

[d]Department of Chemistry, Pennsylvania State University, University Park, Pennsylvania, 16802, United State

[*]Corresponding authors: E-Mail: sylvie.roke@epfl.ch; psc11@psu.edu

Contents

S1. Experimental methods.

S2. Converting measured SF intensity to interfacial $\left|\Gamma^{(2)}\right|^2$ spectra.

S3. SFS O-D spectra.

Supporting Figures:

Figure S1: Measured IR and SF spectra and converted SFS water spectrum.

Figure S2: The correction factor of the IR adsorption correction procedure.

Figure S3: Parameter data of the IR adsorption correction procedure.

Figure S4: Average spectra in Figs. 3 and 4 with error bar.

Supporting Tables:

Table S1: Droplet size distributions for the data plotted in Figs. 2-4.

Table S2: Mobility and zeta potential values for different droplets.

Table S3: Parameters used to fit the spectra in Fig. 2C.

Table S4. FWHM and its error bar of O-D spectra in Figs. 4C and 4D.

S1. Experimental methods.

Electrokinetic mobility measurements. The electrophoretic mobility measurements were performed using laser Doppler velocimetry and phase analysis light scattering, employing a dynamic light scattering instrument (Malvern ZS nano-sizer). To perform the electrophoretic mobility measurements, the nanoemulsions were diluted to 0.05 vol%. The electrophoretic mobility (μ) values were converted into ζ-potential (ζ) values using the following expression: $\mu = \frac{\epsilon_0 \epsilon \zeta f(\kappa R)}{\eta}$ where ϵ_0 is the vacuum permittivity, ϵ is the relative permittivity and η is the viscosity of the bulk phase, $f(\kappa R)$ is Henry's function, κ is the inverse Debye length, and R is the radius of the droplet. We use a more generalized form proposed by Oshima[1] for Henry's equation.

$$f(\kappa R) = \frac{2}{3}[1 + \frac{1}{2\left\{1+\frac{2.5}{\kappa R(1+2e^{-\kappa R})}\right\}^3}] \quad \text{(S1)}$$

Table S1. Droplet size distributions for the data plotted in Figs. 2-4. All samples are prepared with 10 mM Span80 in the oil phase.

Sample	Z-average diameter (radius), nm	PDI
water-in-oil, D_2O : H_2O=100:0	179 (89.5)	0.13
water-in-oil, D_2O : H_2O=85:15	184 (92)	0.18
water-in-oil, D_2O : H_2O=50:50	182 (91)	0.23
water-in-oil, D_2O : H_2O=15:85	172 (86)	0.17
oil-in-water	216 (108)	0.25

Table S2. Mobility and zeta potential values for different droplets.

Sample	Mobility, x10^{-8} m^2/Vs	Zeta Potential, mV
Span80 water-in-oil	-0.01±0.026	-28.7±74.5
Span80 oil-in-water	-1.92±0.027	-45.9±0.6
Bare oil-in-water	-2.37±0.03	-56.6±0.7

Vibrational SFS spectra acquisition. C-H and O-D modes were measured. The acquisition time for a single C–H mode spectrum was 600 s. Measured sum frequency spectra were normalized by VIS and IR pulse energies, acquisition time, and the droplet radii. The C–H stretching spectra were fitted using Levenberg-Marquadt iterations using the equation

$$I_{SFS}(\omega_{IR},\theta) \propto \left|A_{NR} f(\omega_{IR}) e^{i\varphi_{NR}} + \sum_v \frac{A_v(\theta)\gamma_v}{\omega_{IR}-\omega_v+i\gamma_v}\right|^2, \quad \text{(S2)}$$

where A_{NR}, $f(\omega_{IR})$ and φ_{NR} are the amplitude, shape and phase of non-resonant background, $A_v(\theta)$, ω_v, and γ_v denote the amplitude, frequency and linewidth of resonant vibrational modes. Table S3 shows the parameters used to fit the C-H spectra in Fig. 2C.

Table S3. Parameters used to fit the spectra in Fig. 2C.

		Water droplets		Oil droplets	
	ω_v	γ_v	A_v	γ_v	A_v
d^+	2852	14	0.080	15	0.145
r^+	2875	15	0.064	8	0.010
d^-	2920	15	-0.004	22	-0.010
r^-	2965	30	0.089	30	0.059
d^{+FR}	2902	15	-0.167	15	-0.099
r^{+FR}	2935	22	0.029	22	0.008

For the O-D stretching modes, broadband IR pulses between 2200 and 3000 cm^{-1} spaced with 100 cm^{-1} frequency steps were used to probe the entire O-D stretching region. To obtain the interfacial response, prior to size normalization, we used a procedure to correct for IR absorption by the main aqueous phase. This procedure was first reported in Ref. 2, and refined for other solvents in Ref. 3. The infrared spectral profiles were recorded as the sum frequency intensity from a solid film of $BaTiO_3$ nanoparticles and are shown in Fig. S1A and computed $I_0(\omega_{IR})$ is shown in Fig. S1B. The measured sum frequency spectrum at each frequency range was background subtracted and then normalized with IR and VIS pulse energy and acquisition time. The total SF intensity at each pulse center frequency ω_{IR} was computed as a weighted sum:

$$I_{SF}(\omega = \omega_{IR}) = \frac{\sum_{i=1}^{n} I_{SF}^{i}(\omega_{IR}) * I_0^{i}(\omega_{IR})}{\sum_{i=1}^{n} I_0^{i}(\omega_{IR})} \quad \text{(S3)},$$

where the index i runs through all IR excitations that contribute to the intensity at $\omega = \omega_{IR}$ and $I_0^i(\omega_{IR})$ is the incident IR intensity that generates sum frequency signal at $\omega = \omega_{IR}$. The resultant spectrum after the summation is shown in Fig. S1C, which is one set of raw data used to calculate the average spectrum from 2 vol% d-C16 droplets with 10 mM Span80 in D_2O in Fig. 2B (black trace).

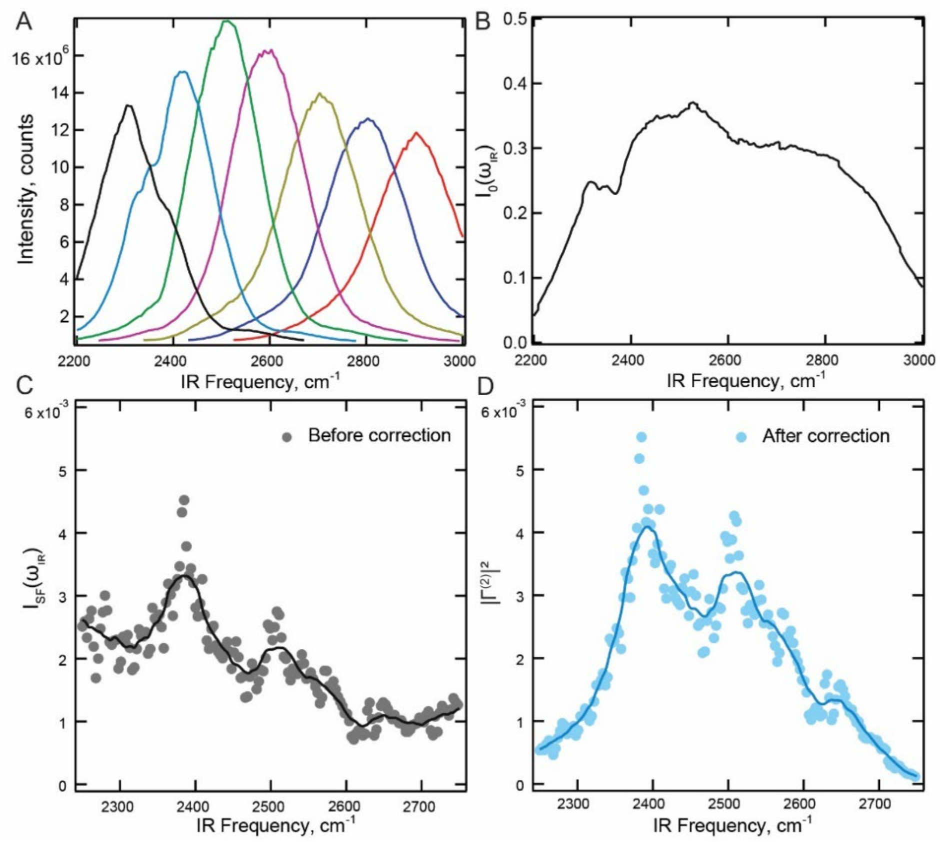

Figure S1: **Measured IR and SF spectra and converted SFS water spectrum**. Procedure for retrieving the interfacial water spectrum of 2% d-C16 droplets with 10 mM Span80 in D_2O. A: The SFS signal from $BaTiO_3$ nanoparticle film, representing the shape of IR pulses used for SFS measurements, as well as B: the computed ($I_0(\omega_{IR})$). C: The total summed SFS spectrum (Eq. S3) for the entire O-D stretch region. D: The resultant $\left|\Gamma^{(2)}\right|^2$ SF spectrum of Span80 d-C16 droplets in 100% D_2O retrieved by dividing the SF measurement (C) by the function $C(\omega_{IR})$ (Fig. S2B, purple trace). The spectra in Fig. S1C and S1D are normalized to unit area and the solid lines represent the running average as guides to the eye. Note that the running average presented here is different from that in Fig. 3 in the main text, where the broader average is used to prevent overinterpretation of the wiggles.

S2. Converting the measured SF intensity to interfacial $\left|\Gamma^{(2)}\right|^2$ spectra.

Recently, we determined the appropriate light-matter interactions and devised a method to retrieve the actual surface response ($\left|\Gamma^{(2)}(\omega_{SF};\omega_{VIS},\omega_{IR})\right|^2$) from the measured SF spectral intensity ($I_{SF}(\omega_{SF};\omega_{VIS},\omega_{IR})$):[4]

$$\left|\Gamma^{(2)}(\omega_{SF};\omega_{VIS},\omega_{IR})\right|^2 \propto \frac{I_{SF}(\omega_{SF};\omega_{VIS},\omega_{IR})}{\int_0^L I_{IR}(\omega_{IR},z) I_{VIS}(\omega_{VIS}) f_{focal}(z)\rho(z)\,dz} = \frac{I_{SF}(\omega_{IR})}{C(\omega_{IR})} \qquad \text{(S4)},$$

where ω_{SF}, ω_{VIS}, and ω_{IR} are the frequencies of the sum frequency, visible, and IR pulses.

$I_{SF}(\omega_{SF};\omega_{VIS},\omega_{IR}) = I_{SF}(\omega_{IR})$ is the measured SF intensity (Fig. S1C). $I_{IR}(\omega_{IR}, z)$ is the intensity of the incident infrared illumination along the optical z axis which is given by Lambert-Beers' law, $I_{IR}(\omega_{IR}, z) = I_0(\omega_{IR})e^{-\alpha(\omega_{IR})z}$, with $I_0(\omega_{IR})$ the incident IR intensity (Fig. S1B) and $\alpha(\omega_{IR})$ the absorption spectrum. $\alpha(\omega_{IR})$ is computed from the measured IR transmission spectrum (T, Fig. S2A and S2C) as $\alpha(\omega_{IR}) = \frac{-ln\,(T(\omega_{IR}))}{L}$. $I_{VIS}(\omega_{VIS})$ is the intensity of the incident visible illumination, $f_{focal}(z)$ is a measured collection function that describes the efficiency of light collection along the optical axis z of the collection optics (Fig. S3), and $\rho(z)$ is the number density of particles at a specific depth, assumed to be uniform (i.e., $\rho(z) = 1$). $C(\omega_{IR})$ is the final correction factor resulting from numerically integrating the denominator in the middle of Eq. S4. Figure S2B (S2D) shows the $C(\omega_{IR})$ factors computed using Eq. S4 from the measured IR transmission spectra in Fig. S2A (S2C). $\Gamma^{(2)}(\omega_{SF};\omega_{VIS},\omega_{IR}) = \Gamma^{(2)}$ is the effective second-order particle susceptibility that describes the spectral interfacial response of droplets dispersed in solution. $\Gamma^{(2)}$ is a function of the scattering angle (θ, defined as the angle between the scattered SF wavevector and that of the phase-matched direction), the average radius (R) of the droplets, and the second-order surface susceptibility ($\chi^{(2)}$).[5-7]

Eq. S4 contains a number of parameters that were experimentally determined. The absorption coefficient $\alpha(\omega_{IR})$ was computed from transmission spectra of H_2O and D_2O water mixtures (Fig. S2A) and water-in-oil droplets (Fig. S2C). Transmission spectra were recorded on a Bruker Vertex 70 FTIR spectrometer, using CaF_2 and quartz windows with an optical path length of 10 microns (bulk water mixtures) or 200 microns (water-in-oil emulsions). Note that the water spectra from oil-in-water droplets were corrected using the transmission spectra of bulk water (H_2O and D_2O mixtures) since it was the main phase for the oil droplets and contributed to significant absorption. On the other hand, for water-in-oil droplets, the water spectra were corrected using the transmission spectrum of the water-in-oil emulsions to take into account the main phase as well as the minor phase of water.

Another parameter is $f_{focal}(z)$, the collection function that describes the efficiency of light collection along the optical axis z of the collection optics. This function was measured using a sample of stearyl covered silica particles (R=123 nm) dispersed in CCl_4.[8] By recording the SFS spectrum while translating the sample along the optical axis (see also Ref. 9), $f_{focal}(z)$, was determined. Figure S3 shows the integrated spectral intensities and the fit function that was used as $f_{focal}(z)$. The measured SF intensity and the corrected $\left|\Gamma^{(2)}\right|^2$ spectra are shown in Figs. S1C and S1D, respectively, which are the raw and processed data of a sample having 2 vol% d-C16 droplets with 10 mM Span80 in 100% D_2O, measured with the SSP polarization combination.

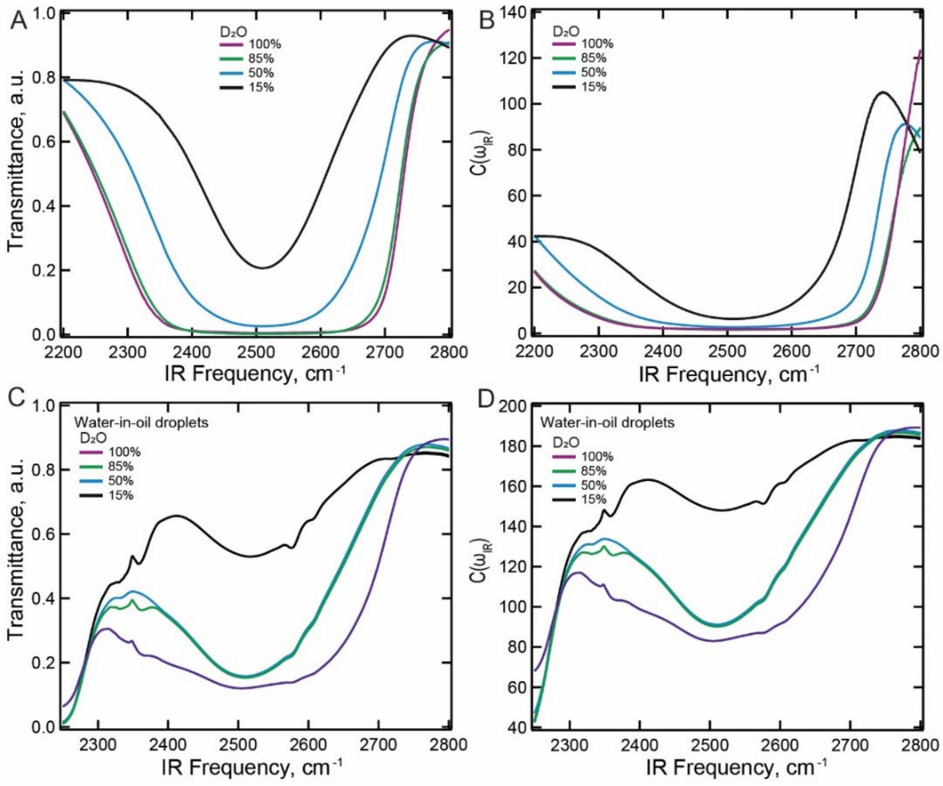

Figure S2: The correction factor of the IR adsorption correction procedure. A: FTIR transmittance profiles of D_2O: H_2O mixtures with 10 μm path length measured using sample cells similar to the ones used for SFS measurements. B: The correction factor resulting from numerically integrating the denominator in the middle part of Eq. S4 using transmittance profiles in A, which represents the spectral distortions due to linear absorption of IR light. C: FTIR transmittance profiles of water-in-oil emulsions with a 200 μm path length using sample cells similar to the ones used for SFS measurements. D: The correction factor resulting from numerically integrating the denominator in the middle part of Eq. S4 using transmittance profiles in C.

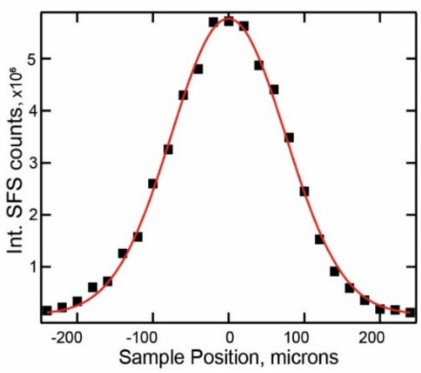

Figure S3: Input data for the IR adsorption correction procedure. The integrated spectral intensities are plotted as a function of sample position. The red line is a fit that was used for the $f_{focal}(z)$ function in Eq. S4.

S3. SFS O-D spectra.

Averaged O-D spectra with error bar. The vibrational SFS spectra from 100% D_2O oil-in-water and water-in-oil droplets shown in Fig. 3B and 15% D_2O oil-in-water and water-in-oil droplets shown in Fig. 4 are the average from 3 individual measurements. The corresponding averaged spectra are replotted with error margins (colored range) in Figs. S4A and 4B respectively for oil-in-water and the water-in-oil droplets.

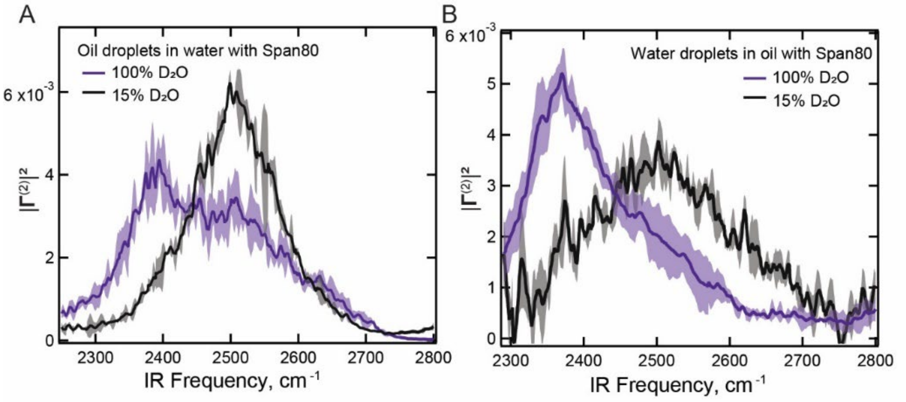

Figure S4: Average spectra in Figs. 3 and 4 with error bar. A: The average spectra (solid line) with error bar (shade) from d-C16 droplets in 100% D_2O (purple) and 15% D_2O (black) with 10 mM Span80. B: The average spectra (solid line) with error bar (shade) from 100% D_2O droplets (purple) and 15% D_2O droplets (black) in d-C16 with 10 mM Span80.

Spectral bandwidths. Figure 4F shows the full-width-at-half-maximum (FWHM) values for isotope diluted water spectra for oil droplets in water (Fig. 4C) and water droplets in oil (Fig. 4D). Note that all water spectra shown in Figs. 4C and 4D exhibit distinct shapes, varying from one asymmetric peak to a two-peak-like profile, which cannot be suitably fitted by either a classical Gaussian or Lorentzian. Hence, the FWHM values are estimated by determining the spectral full width at half maximum intensity, which are listed in Table S4. The error bars are from the uncertainty that is mainly due to elevated baseline (induced by the IR absorption of the deuterated hexadecane) and the asymmetric spectral line shape.

Table S4. FWHM and its error bar of O-D spectra in Figs. 4C and 4D.

	Oil droplets in water		Water droplets in oil	
	FWHM, cm^{-1}	Error bar	FWHM, cm^{-1}	Error bar
15% D_2O	140	8.5	211	11.1
50% D_2O	222	13	213	15.3
85% D_2O	242	6.1	199	10.4
100% D_2O	255	8.3	194	12.1

References

(1) Ohshima, H. A Simple Expression for Henrys Function for the Retardation Effect in Electrophoresis of Spherical Colloidal Particles. *J. Col. Sci.* **1994**, *168* (1), 269-271.

(2) Pullanchery, S.; Kulik, S.; Rehl, B.; Hassanali, A.; Roke, S. Charge Transfer Across C-H···O Hydrogen Bonds Stabilizes Oil Droplets in Water. *Science* **2021**, *374* (6573), 1366-1370.

(3) Pullanchery, S.; Zhang, L.; Kulik, S.; Roke, S. Interfacial Inversion, Interference, and IR Absorption in Vibrational Sum Frequency Scattering Experiments. *J. Phys. Chem. B* **2023**, *127* (30), 6795-6803.

(4) Kulik, S.; Pullanchery, S.; Roke, S. Vibrational Sum Frequency Scattering in Absorptive Media: A Theoretical Case Study of Nano-objects in Water. *J. Phys. Chem. C* **2020**, *124* (42), 23078-23085.

(5) Roke, S.; Bonn, M.; Petukhov, A. V. Nonlinear Optical Scattering: The Concept of Effective Susceptibility. *Phys. Rev. B* **2004**, *70* (11).

(6) De Beer, A. G. F.; Roke, S. Sum Frequency Generation Scattering from the Interface of an Isotropic Particle: Geometrical and Chiral Effects. *Phys. Rev. B* **2007**, *75* (24).

(7) Dadap, J. I.; de Aguiar, H. B.; Roke, S. Nonlinear Light Scattering from Clusters and Single Particles. *J. Chem. Phys.* **2009**, *130* (21).

(8) Roke, S.; Berg, O.; Buitenhuis, J.; van Blaaderen, A.; Bonn, M. Surface Molecular View of Colloidal Gelation. *P. Natl. Acad. Sci. USA* **2006**, *103* (36), 13310-13314

(9) Roke, S.; Roeterdink, W. G.; Wijnhoven, J. E. G. J.; Petukhov, A. V.; Kleyn, A. W.; Bonn, M. Vibrational Sum Frequency Scattering from a Submicron Suspension. *Phys. Rev. Lett.* **2003**, *91* (25).
